# Echocardiographic assessment of left ventricular diastolic dysfunction in congenital diaphragmatic hernia

**DOI:** 10.1038/s41390-026-05029-w

**Published:** 2026-04-20

**Authors:** Spandana Rupa Madabhushi, Gergely Toldi

**Affiliations:** 1 Neonatal Intensive Care Unit, Wellington Regional Hospital, Wellington, New Zealand; 2 Liggins Institute, University of Auckland, Auckland, New Zealand

Congenital diaphragmatic hernia (CDH) continues to have significant mortality despite advances in prenatal and postnatal management. Cardiac dysfunction results from aberrant pulmonary and cardiac development following herniation of abdominal viscera into the intrathoracic cavity, with or without further underlying congenital structural heart disease. The degree of cardiac dysfunction is an important determinant of CDH disease severity and outcome.^1^

Cardiac dysfunction can be further characterised as left ventricular (LV) or right ventricular (RV) dysfunction, as well as systolic or diastolic. Early (i.e. within 48 h) LV dysfunction in isolation, or in combination with RV dysfunction, has been associated with increased mortality in infants with CDH.^2^ Diastolic dysfunction is harder to determine as it relies on active ventricular relaxation, elastic recoil and chamber stiffness.^3^ Moenkemeyer et al. have described the detrimental effect early RV diastolic dysfunction can have on morbidity, but thus far, LV diastolic dysfunction has not been investigated in CDH.^4^ Although the gold standard for assessing diastolic function is through cardiac conductance catheterisation, this invasive technique is not a practical solution for the neonatal population.

In this single-centre, retrospective observational cohort study, Cantone et al. aim to assess the feasibility of using clinical echocardiography to evaluate early LV diastolic dysfunction and how this contributes to pulmonary hypertension and CDH disease severity.^5^ The authors assessed LV diastolic function by using echocardiography parameters recommended by the European Association of Cardiovascular Imaging and the American Society of Echocardiography.^6^ The following parameters were included in the analysis: pulsed-wave mitral valve inflow velocity, pulsed-wave tissue Doppler imaging in the left ventricular free wall and left atrial reservoir strain. Table 1 summarises some basic considerations in relation to the acquisition and interpretation of these parameters.

**Table 1:** Considerations in relation to the acquisition and interpretation of left ventricular diastolic function parameters in neonates with congenital diaphragmatic hernia.

Parameter	Acquisition	Interpretation
Pulsed-wave mitral valve inflow velocity^6^	1. Apical 4-chamber view 2. Colour Doppler 3. Sample gate positioned just beyond mitral valve leaflet tips (Minimise angle of insonation to <20°) 4. Measure peak early (E) and late (A) velocities 5. Express as E/A ratio	• Peak early (E) velocity: ventricular filling due to relaxation, by measuring flow through the mitral valve • Late (A) velocity: ventricular filling due to atrial contraction by measuring flow through the mitral valve Reduced E/A ratio indicates diastolic dysfunction: • Impaired ventricular relaxation affects passive filling (smaller E) • Larger volume in atria to eject (larger A)
Pulsed-wave tissue Doppler imaging (TDI) at the mitral annulus^6^	1. Apical 4-chamber view 2. Colour Doppler 3. Sample Gate positioned at mitral annulus (lateral aspect) 4. Obtain peak longitudinal myocardial velocity in: • Systole (LV s’) • Early Diastole (LV e’): Ventricular filling due to relaxation at the mitral annulus • Late Diastole (LV a’): Ventricular filling due to atrial contraction, measured at the mitral annulus	Calculate E:e’ ratio to estimate left ventricular end-diastolic pressure (LVEDP) Diastolic dysfunction increases E:e’ ratio (impaired ventricular relaxation leads to smaller e’ and larger E waves)
Left atrial strain^7^	1. Apical 4-chamber view (uses speckle-tracking echocardiography) 2. Software tracks speckle pattern in left atrial wall length across cardiac cycle, producing a strain curve 3. Correlates with ECG, uses QRS onset as a surrogate marker of end-diastole	Left atrial reservoir strain (LASr): • Maximum relaxation of the atrial wall during ventricular contraction • Receives blood from pulmonary veins Left Atrial Conduit Strain (LAcd): • Passive atrial shortening after the mitral valve opens • Ventricle relaxes, and the LA acts as a conduit between the pulmonary veins and the LV Left Atrial contraction strain (LAct): • LA contracts and shortens at the end of diastole

Cantone et al. report on a retrospective analysis of data collected in infants >32 weeks with either prenatally or postnatally detected CDH.^5^ Infants were included if they received an echocardiogram within the first 24 h and did not have significant, life-limiting associated anomalies, including major chromosomal variants. Alongside demographic data, CDH characteristics such as the lung to head ratio, defect size and defect position were reviewed. The clinical outcomes reported include survival to discharge, use of extra-corporeal membrane oxygenation, age at surgery and ongoing requirement for invasive ventilation at 14 days of life.

The authors analysed a total of 52 infants, with 77% of infants born ≥37 weeks, and 90% having a prenatal diagnosis. All cases were managed according to established protocols, which included delivery room intubation, early escalation to high frequency oscillation ventilation and echocardiography to guide cardio-respiratory management. Normative echocardiographic parameters collected from healthy newborns (i.e. not from a study-specific control group) were compared to the CDH cases and then used to define reduced diastolic function.^7^

The authors report that over 70% of infants with CDH had early LV diastolic dysfunction. Correlation analyses were undertaken comparing different parameters of LV diastolic function to outcome measures. Of note, although statistically significant, most of the correlations reported were modest, with *r*^2^ < 0.4. One parameter, left atrial reservoir strain (LASr), which describes the ability of the left atrium to stretch during ventricular contraction, was found to be lower in non-surviving infants and those who remained invasively ventilated at >14 days of life. This is in keeping with similar studies assessing LASr.^8^

When comparing LASr to measures of gas exchange, the authors identified that reduced LASr was associated with higher maximal carbon dioxide levels and increased FiO2. They hypothesised that reduced diastolic function contributed to elevated pulmonary venous pressures, leading to pulmonary venous congestion and more difficult ventilation. Whilst there was some correlation seen between echocardiographic markers of LV diastolic function and pulmonary artery pressures, the effect was not significant.

Reduced LASr suggests raised left atrial pressures and increased ventricular stiffness, as the overworked ventricle becomes stiffer and makes the atria work harder to pump blood. However, in the neonatal population, the presence of shunts at both the atrial and ductal levels can lead to volume loading and also reduce LASr. As such, the presence and the direction of shunts should be considered when evaluating LV diastolic function. Additionally, thought should be given to other transitional changes unique to the newborn, including changes in pulmonary and systemic vascular resistance. This reiterates the importance of using more than one measure in functional cardiac assessments.

The assessment of these parameters could be affected by differences in ultrasound devices and post-processing software, especially for the LASr measurements, which require speckle-tracking echocardiography. This specialised software must include functions to assess the left atrium to be applied clinically. As a relatively new parameter, neonatal LASr measurements will require standardised image acquisition. Smaller studies within the neonatal population do show some inter-operator variability when acquiring these measurements.^7^ Thus, these factors and operator experience may limit the value and utility of these parameters in the clinical setting.

Future work would need to validate these findings in a larger, multi-centre cohort, with a study-specific control group. As the authors mention, there may also be value in the simultaneous assessment of pulmonary vein velocities and left atrial volume to better evaluate the effects of LV diastolic dysfunction.

Ultimately, these findings conclude that early echocardiographic assessment of LV diastolic dysfunction is feasible in neonates with CDH. Quantitative and sequential assessment of LV diastolic dysfunction will enable tailored management strategies, including a more informed choice of therapeutic strategy, overall contributing to improved patient outcomes.
